# GDF11/BMP11 activates both smad1/5/8 and smad2/3 signals but shows no significant effect on proliferation and migration of human umbilical vein endothelial cells

**DOI:** 10.18632/oncotarget.7642

**Published:** 2016-02-23

**Authors:** Yong-Hui Zhang, Feng Cheng, Xue-Ting Du, Jin-Lai Gao, Xiao-Lin Xiao, Na Li, Shan-Liang Li, De-Li Dong

**Affiliations:** ^1^ Department of Pharmacology (The State-Province Key Laboratories of Biomedicine-Pharmaceutics of China, Key Laboratory of Cardiovascular Research, Ministry of Education), Translational Medicine Research and Cooperation Center of Northern China, Heilongjiang Academy of Medical Sciences, Harbin Medical University, Harbin, P.R.China

**Keywords:** bone morphogenetic protein 11, growth differentiation factor 11, smad2/3, smad1/5/8, endothelial cells, Pathology Section

## Abstract

GDF11/BMP11, a member of TGF-β superfamily, was reported to rejuvenate heart, skeletal muscle and blood vessel architecture in aged mice. However, the rejuvenative effects of GDF11 were questioned recently. Here, we investigated the effects of GDF11 on smad and non-smad signals in human umbilical vein endothelial cells (HUVECs) and the effects of GDF11 on proliferation and migration of HUVECs and primary rat aortic endothelial cells (RAECs). GDF11 factor purchased from two different companies (PeproTech and R&D Systems) was comparatively studied. Western blot was used to detect the protein expressions. The cell viability and migration were examined by using MTT and wound healing assays. Results showed that GDF11 activated both smad1/5/8 and smad2/3 signals in HUVECs. GDF11 increased protein expression of NADPH oxidase 4(NOX4) in HUVECs. GDF11 showed no significant effect on the protein level of p38, p-p38, ERK, p-ERK, Akt, p-Akt (Ser473) and p-Akt(Thr308), but increased the protein level of p-JNK and p-AMPK in HUVECs, and these increases were inhibited by antioxidant mitoTEMPO treatment. GDF11 slightly increased cell viability after short-term treatment and slightly decreased cell viability after long-term treatment. GDF11 showed no significant effect on cell proliferation and migration. These data indicated that the notion of GDF11 as a rejuvenation-related factor for endothelial cells needs to be cautious.

## INTRODUCTION

Growth differentiation factor 11(GDF11), also known as bone morphogenetic protein 11(BMP11), was first cloned and characterized from rat incisor pulp RNA as a member of bone morphogenetic protein/transforming growth factorβ (BMP/TGF-β) superfamily [1]. Like other members of the TGF-β superfamily, GDF11 binds to type II receptors, and then the complex recruits activin type I receptors ALK4, ALK5 and ALK7 to activate smad2/3 proteins (R-smads) pathway, which associates with common mediator smad4. The smad protein complex translocates into the nucleus and interacts with transcriptional co-activators or co-repressors to regulate promoter activity to positively or negatively control gene expression.

GDF11 not only contributes to embryonic development and histogenesis, but also plays a role in metabolic disorders, and cancers [2, 3]. Recently, several noticeable studies showed that GDF11 rejuvenated heart and skeletal muscle in aged mice [4], and GDF11 increased proliferation of brain capillary endothelial cells, migration of endothelial progenitor cells and improved the vascular and neurogenic rejuvenation of the aging mouse brain [5, 6]. However, the rejuvenative effects of GDF11 were questioned by other studies. These reports doubted the decrease of circulating GDF11 level in aging muscle, heart, and brain phenotypes [7, 8], and restoring GDF11 in old mice had no effect on cardiac structure or function [9]. Besides, GDF11/BMP11 had deleterious effects on aging skeletal muscle [8]. Thus, it is essential to investigate the effects of GDF11 on endothelial cells, which correlate with multiple cardiovascular diseases.

Since GDF11 belongs to BMP/TGF-β superfamily, the canonical signals induced by BMP/TGF-β superfamily should be the basis of GDF11 action. Therefore, in the present work, we firstly identified the effects of GDF11 on smad and non-smad signals in human umbilical vein endothelial cells (HUVECs), and then studied the effects of GDF11 on cell viability, proliferation and migration of HUVECs. In order to ensure the reliability of results, we used GDF11 factor purchased from two different companies (PeproTech and R&D Systems) to treat HUVECs and primary rat aortic endothelial cells (RAECs). We found that GDF11 activated both smad1/5/8 and smad2/3 signals but showed no significant effects on proliferation and migration of HUVECs.

## RESULTS

### GDF11 activates both smad1/5/8 and smad2/3 signals in HUVECs

**Figure 1:**
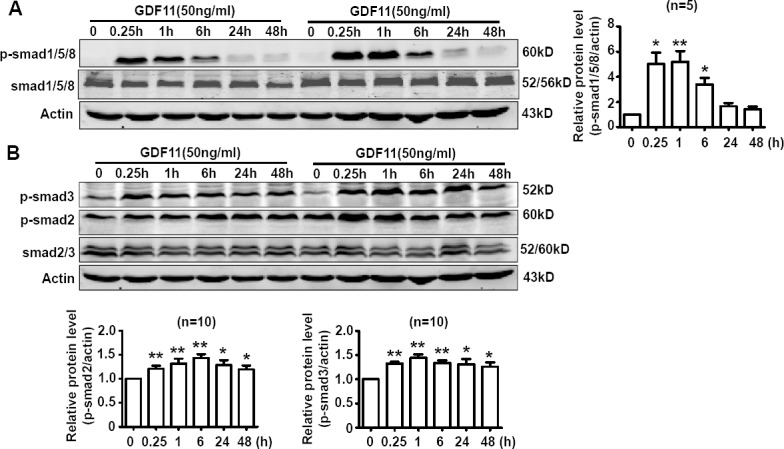
GDF11 activates both smad1/5/8 signal and smad2/3 signals in HUVECs **A.** The time-course of GDF11-induced smad1/5/8 activation in HUVECs. *P < 0.05 *vs*. control (0h), **P < 0.01 *vs*. control (0h). *n* = 5. **B.** The time-course of GDF11-induced smad2/3 activation in HUVECs. *P < 0.05 *vs*. control (0h), **P < 0.01 *vs*. control (0h). *n* = 10.

**Figure 2:**
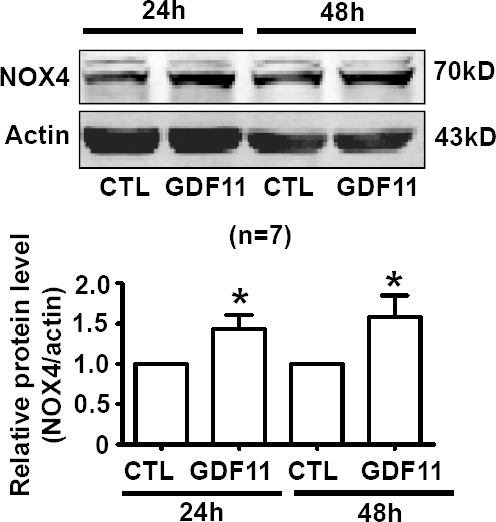
GDF11 increases NOX4 protein expressions NOX4 protein level was increased after GDF11 (50ng/ml) treatment for 24h and 48h in HUVEC cells. *P < 0.05 *vs*. control. *n* = 7.

Activating smad1/5/8 is the typical action of BMP members [10] and activating smad2/3 is the typical action of TGF-β members [11]. We speculated that GDF11 should have the properties of both BMPs and TGF-β, so we firstly examined the effects of GDF11 on smad1/5/8 and smad2/3 signals in HUVECs. As shown in Figure 1A and 1B, GDF11 significantly activated both smad1/5/8 and smad2/3 signals in HUVECs. GDF11 treatment did not affect the total smad2/3 and total smad1/5/8 expressions in protein level (data not shown). The time-course of smad1/5/8 and smad2/3 activation by GDF11 treatment was different. For smad1/5/8 signal, the peak of GDF11-induced smad1/5/8 activation appeared at 0.25h after treatment; with the time lapsing, the activation declined and disappeared after 24h. For smad2/3 signal, the activation by GDF11 was constant during the treatment time from 0 to 48 h. Activation of smad2/3 signaling pathway increased the expression of NOX4 in the endothelial cells, fibroblasts, human pulmonary artery smooth muscle cells and breast cancer cells [12-15] and activation of the smad2/3/NOX4/H_2_O_2_ signaling pathway had a positive role in cell proliferation and cell migration of endothelial cell [15]. Therefore, we tested the effect of GDF11 on NOX4 protein expressions in HUVECs. GDF11 treatment (50ng/ml) increased NOX4 protein level after 24 and 48h treatment (Figure 2). NOX4 induces the production of hydrogen peroxide (H_2_O_2_) and superoxide anion O^2^−^^ [16, 17], suggesting that GDF11 would have ROS-related actions.

### The effects of GDF11 on MAPKs, Akt, and AMPK signals in HUVECs

**Figure 3:**
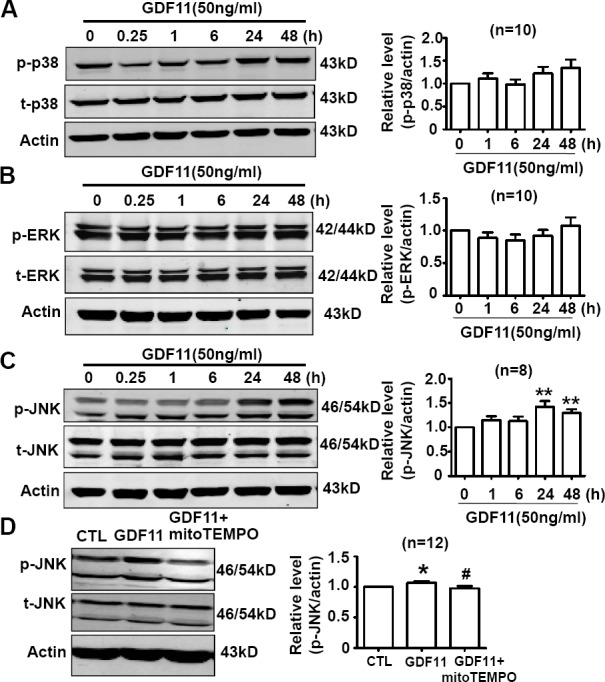
Effects of GDF11 on MAPK signals in HUVECs (**A**.-**B**.) GDF11 had no significant effect on p38 and ERK MAPK signals in HUVECs. *n* = 10 **C.** GDF11 increased the protein level of p-JNK after at GDF11 treatment for 24h and 48h in HUVECs. **P < 0.01 *vs*. control. *n* = 8. **D.** MitoTEMPO inhibited GDF11-induced JNK activation in HUVECs. The cells were pre-treated with mitoTEMPO(25nM) for 1h and then GDF11(50ng/ml) was added. *P < 0.05 *vs*. control. # *P* < 0.05 *vs*. GDF11 treatment group. *n* = 12.

**Figure 4:**
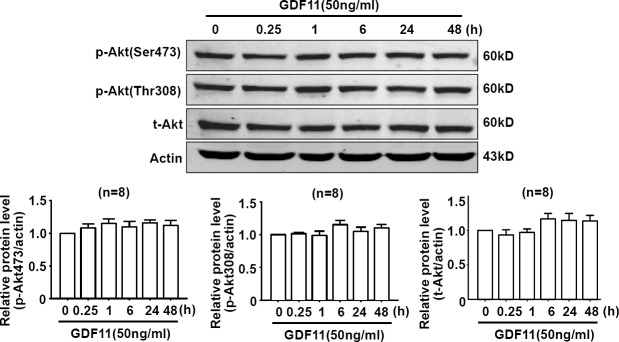
GDF11 has no effect on Akt signal in HUVECS GDF11 had no significant effect on the level of p-Akt(Ser473), p-Akt(Thr308) and total Akt protein following GDF11 stimulation in HUVEC cells. *n* = 8.

**Figure 5:**
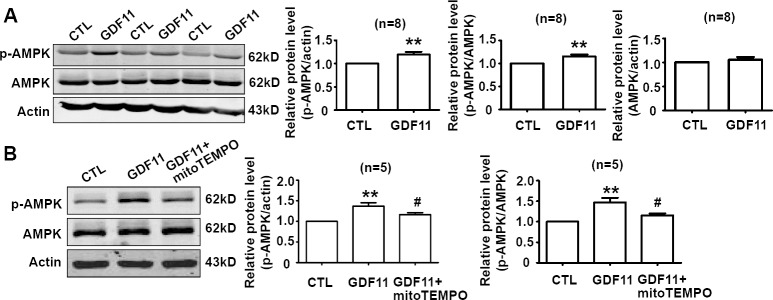
GDF11 induces AMPK activation which is inhibited by mitoTEMPO **A.** GDF11 increased the protein level of p-AMPK after GDF11 treatment (50ng/ml) for 48h in HUVECs. **P < 0.01 *vs*. control. *n* = 8. **B.** MitoTEMPO inhibited GDF11-induced MAPK activation in HUVECs. The cells were pre-treated with mitoTEMPO(25nM) for 1h and then GDF11(50ng/ml) was added. **P < 0.01 *vs*. control.# *P* < 0.05 *vs*. GDF11 treatment group. *n* = 5.

Non-smad pathways are also involved in the biological functions of BMP and TGF-β members [18, 19], then, we examined the effects of GDF11 on MAPKs, Akt and AMPK signals in HUVECs. GDF11 showed no significant effect on the protein levels of p38, p-p38, ERK, and p-ERK during the treatment period from 0 to 48h (Figure 3A and 3B), but increased p-JNK after 24 and 48 h treatment (Figure 3C). Antioxidant mitoTEMPO (25nM) inhibited the GDF11-induced increase of p-JNK expression at 48h (Figure 3D), indicating that GDF11-induced JNK activation was ROS-dependent. GDF11 treatment did not affect total JNK expression in protein level (data not shown). GDF11 showed no effect on the protein levels of Akt, p-Akt (Ser473) and p-Akt (Thr308) during the treatment period from 0 to 48h (Figure 4). GDF11 (50ng/ml) activated AMPK 48h post-treatment and mitoTEMPO (25nM) inhibited GDF11-induced AMPK activation in HUVEC cells (Figure 5A and 5B).

### Effects of GDF11 on cell viability, cell migration and cell proliferation in endothelial cells

**Figure 6:**
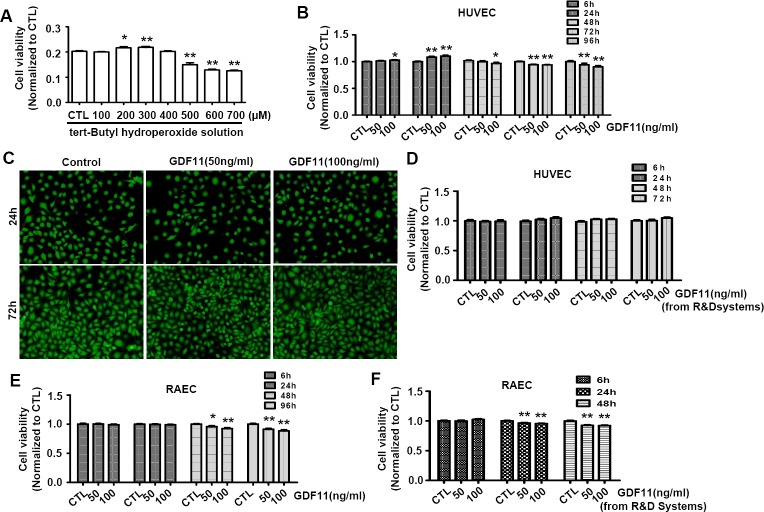
Effects of GDF11 on cell viability in HUVECs and RAECs **A.** Tert-Butyl hydroperoxide increased cell viability at 200μM and 300μM doses and decreased cell viability in the doses from 500 to 700μM after 6h treatment. *P < 0.05 *vs*. control. **P < 0.01 *vs*. control. **B.** GDF11 (50ng/ml) purchased from PeproTech slightly increased cell viability after 24h treatment and slightly decreased cell viability after 72h and 96h treatment in HUVECs. GDF11(100ng/ml) purchased from PeproTech slightly increased the cell viability in HUVEC cells at 6h and 24h and decreased the cell viability at 48h, 72h and 96h. *P < 0.05 *vs*. control. **P < 0.01 *vs*. control. **C.** The representative photos of live and dead cell staining. The live cells were stained with calcein AM in green, and the dead cells were stained with ethidium homodimer-1 in red. **D.** GDF11 purchased from R&D Systems showed no significant effect on cell viability of HUVECs. **E.** GDF11 purchased from PeproTech slightly decreased cell viability after 48h and 96h treatment in aortic endothelial cells (RAECs). *P < 0.05 *vs*. control. **P < 0.01 *vs*. control. **F.** GDF11 purchased from R&D Systems slightly decreased cell viability after 24h and 48h treatment in aortic endothelial cells (RAECs). **P < 0.01 *vs*. control.

It was reported that H_2_O_2_ promoted endothelial cell growth at a low dose and induced cell apoptosis at a higher dose [20]. Firstly, We tested the effects of tert-Butyl hydroperoxide(t-BHP), which was a derivative of H_2_O_2_ and was used as lipid peroxide prototype to induce free radical production [21], on cell viability of HUVECs. The t-BHP-induced changes of cell viability were associated with the t-BHP concentrations. In the range from 200 to 300μM, t-BHP increased the cell viability, but in the range from 500 to 700μM, t-BHP decreased the cell viability (Figure 6A). Then, we examined the effects of GDF11 on cell viability of HUVECs. As shown in Figure 6B, GDF11 slightly increased the cell viability after 24h treatment, and slightly decreased the cell viability after 72 and 96 h treatment. Live- and dead-cell staining assay showed that GDF11 did not induce cell death (Figure 6C). By using GDF11 purchased from another company (R&D Systems), the cell viability was not apparently changed in HUVECs (Figure 6D). We further studied the effects of GDF11 purchased from two different companies (PeproTech and R&D Systems) on cell viability of primary rat aortic endothelial cells (RAECs). Both of GDF11 slightly reduced the cell viability after 48h treatment (Figure 6E and Figure 6F). Comprehensive analysis of above data indicated that GDF11 increased the cell viability after short time treatment but decreased the cell viability after long time treatment. However, whatever the increase or decrease, the extent of changes was too small to be meaningful.

**Figure 7:**
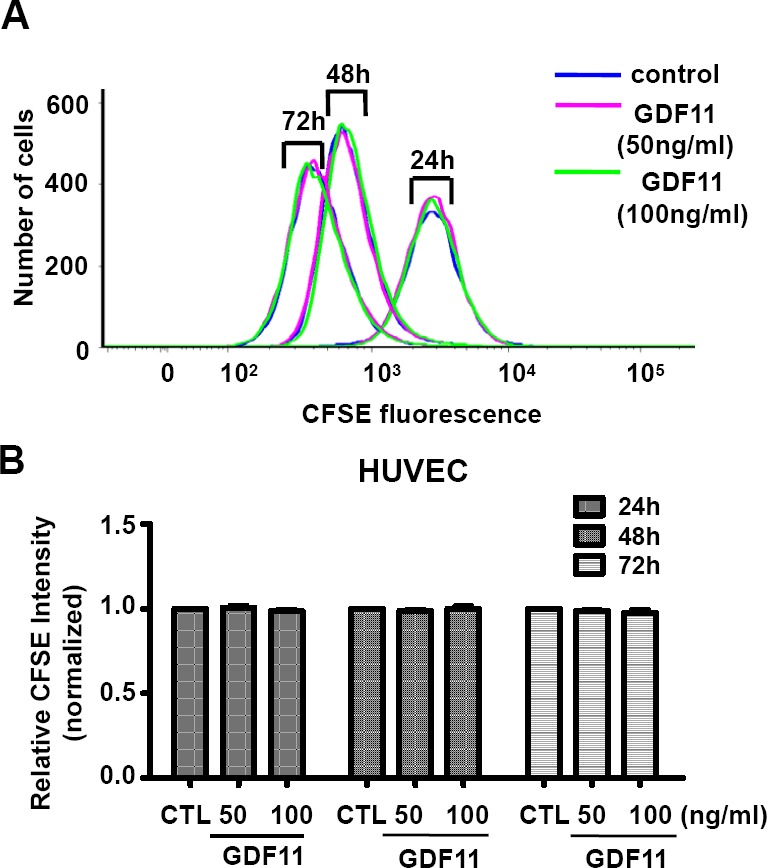
GDF11 shows no significant effect on cell proliferation of HUVECs Cell proliferation was measured by carboxyfluorescein diacetate succinimidyl ester (CFSE) staining and analyzed by flow cytometry. *n* = 6.

**Figure 8:**
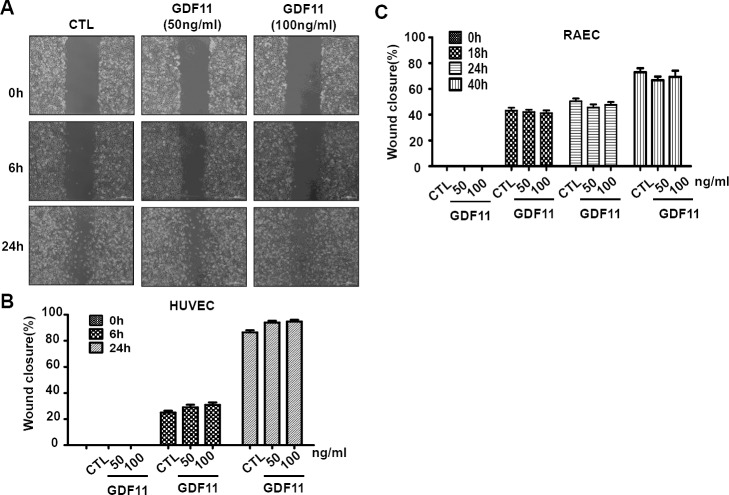
GDF11 shows no significant effect on cell migration in HUVECs and RAECs **A.** The representative photographs of wound closure in HUVECs were shown in **A.** and the summarized data was shown in **B.**. *n* = 43. **C.** GDF11 showed no significant effect on cell migration in RAECs (rat aorta endothelial cells). *n* = 15.

Next, we examined the effects of GDF11 on cell proliferation and cell migration of HUVECs. GDF11 showed no significant effect on cell proliferation and cell migration in HUVECs and RAECs (Figure 7 and Figure 8).

### Effects of GDF11 on eNOS expression in HUVECs

**Figure 9:**
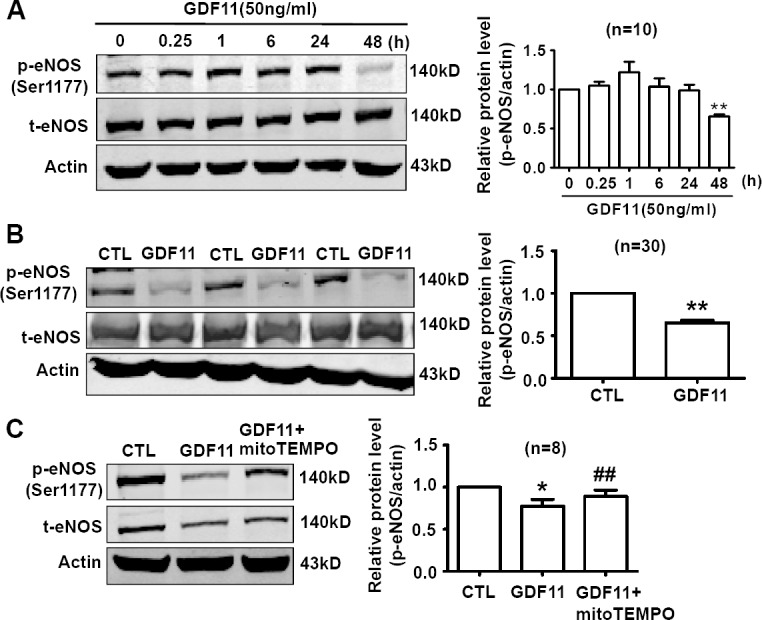
GDF11 decreases the phosphorylation level of eNOS (Ser1177) in HUVECs **A.** The time-course of eNOS expressions showed that GDF11 significantly reduced p-eNOS(Ser1177) expression in protein level after 48h treatment. **P < 0.01 *vs*. 0h. *n* = 10. **B.** Compared with parallel control, GDF11 reduced p-eNOS(Ser1177) expression in protein level after 48h treatment. **P < 0.01 *vs*. Control. *n* = 30. **C.** GDF11-induced decrease of p-eNOS(Ser1177) protein level was restored by antioxidant mitoTEMPO (25nM). The cells were pre-treated by mitoTEMPO for 1h and then GDF11(50ng/ml) was added.*P < 0.05 *vs*. control.## *P* < 0.01 *vs*. GDF11-treated group. *n* = 8.

eNOS plays an important role in maintenance of vascular homeostasis. Inhibition of ROS generation in endothelial cells restored ROS-induced decrease of p-eNOS levels [22]. Since GDF11 induced NOX4 up-regulation, we examined the effects of GDF11 on eNOS expression in HUVECs. As shown in Figure 9A, the time-course of eNOS expressions showed that GDF11 significantly reduced p-eNOS(Ser1177) expression in protein level after 48h treatment. Western blot with parallel control also demonstrated that GDF11 reduced p-eNOS(Ser1177) expression in protein level after 48h treatment (Figure 9B). The decreased p-eNOS(Ser1177) protein level was restored by antioxidant mitoTEMPO, indicating that GDF11-induced down-regulation of p-eNOS was ROS-dependent in HUVECs (Figure 9C).

### Effects of GDF11 on cell viability under serum-deprivation culture condition

**Figure 10:**
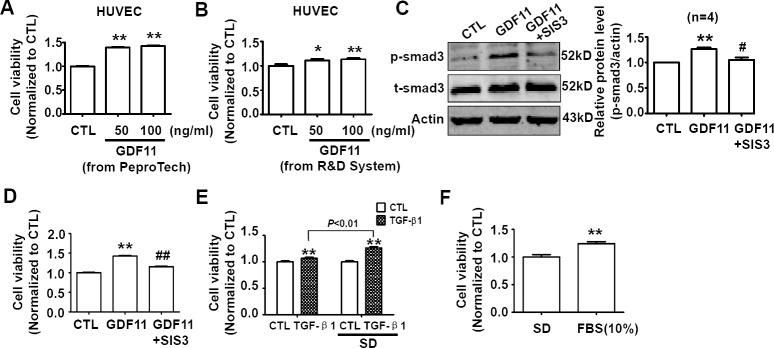
Effects of GDF11 on cell viability of HUVECs in serum-deprivation culture condition **A.** GDF11 (50ng/ml and 100ng/ml) purchased from PeproTech increased cell viability of HUVECs after 24h treatment in serum-deprivation culture condition. **P < 0.01 *vs*. control. *n* = 36. **B.** GDF11 (50ng/ml and 100ng/ml) purchased from R&D Systems increased cell viability of HUVECs after 24h treatment in serum-deprivation culture condition.*P < 0.05, **P < 0.01 *vs*. control. *n* = 12. **C.** SIS3 (5μM) inhibited GDF11-induced smad3 activation in HUVECs. The cells were pre-treated by SIS3 (5μM) for 1h and then GDF11 (50ng/ml) was added. **P < 0.01 *vs*. control. # *P* < 0.05 *vs*. GDF11 treatment group. **D.** SIS3 (5μM) inhibited GDF11-induced increase of cell viability of HUVECs in serum-deprivation culture condition. **P < 0.01 *vs*. control. ## *P* < 0.01 *vs*. GDF11 treatment group. *n* = 36. **E.** TGF-β1 (100ng/ml) increased cell viability in normal and serum-deprivation culture condition after 24h treatment. The effect of increasing cell viability was more significant in serum-deprivation (SD) than in normal culture condition after 24h treatment. **P < 0.01 *vs*. control. *n* = 24. **F.** Fetal bovine serum (10%) supplement increased cell viability comparing with serum-deprivation condition. **P < 0.01 *vs*. control. n = 12. FBS, fetal bovine serum.

The serum-deprivation cell culture condition is mostly used in the studies on cytokines [23]. It should be noticed that serum-deprivation significantly decreased the cell viability, and serum-deprivation induced apoptosis is observed in a variety of cells including endothelial cells [24-27]. Furthermore, the serum-deprivation condition can not mimic any conditions *in vivo*. Therefore, we did not think that the serum-deprivation was a reasonably experimental condition for GDF11 study. Nevertheless, GDF11 was reported to increase proliferation of brain capillary endothelial cells in serum-free basal medium previously [5], we also examined the effects of GDF11 in serum-deprivation culture condition. After GDF11 (from PeproTech and R&D Systems) treatment, cell viability increased significantly in serum-deprivation condition after 24h treatment (Figure 10A and 10B). In serum-deprivation condition, GDF11 still activated smad3 which was inhibited by smad3 inhibitor SIS3 (5μM) (Figure 10C), and SIS3 inhibited GDF11-induced increase of cell viability (Figure 10D). We also found that TGF-β1 and fetal bovine serum supplement had the similar effects of increasing cell viability as GDF11 in serum-deprivation culture condition (Figure 10E and 10F), indicating that the effect of GDF11 was not unique.

## DISCUSSION

Identifying the molecular mechanism of rejuvenation is important for developing therapies to treat age-related diseases. Recently, GDF11/BMP11 was suggested as an anti-aging agent. GDF11 not only rejuvenated heart and skeletal muscle in aged mice [4], but also increased proliferation of brain capillary endothelial cells and improved the vascular and neurogenic rejuvenation of the aging mouse brain [5]. However, recent studies questioned this opinion and reported a potent inhibitory effect of GDF11 on skeletal muscle regeneration [8]. Then, a series of studies showed that GDF11 had no positive role in rescuing aging-related pathological hypertrophy and the level of circulating GDF11 in aging animals was not decreased [7-9]. Because GDF11 was reported to increase proliferation of brain capillary endothelial cells and improve blood vessel architecture, we hypothesized that GDF11 would increase the cell viability, proliferation and migration of human umbilical vein endothelial cells. Therefore, we investigated the effects of GDF11 on smad and non-smad signals, cell viability, proliferation and migration in HUVECs. In the present study, we found that GDF11 activated both smad1/5/8 and smad2/3 signals, showed slight effect on cell viability, but had no significant effect on the proliferation and migration in HUVECs under complete culture medium containing serum.

In our study, we adopted complete medium containing serum to culture the cells. Katsimpardi L *et al.* found that GDF11 increased proliferation of primary brain capillary endothelial cells under serum deprivation condition, which was consistent with our results that GDF11 increased cell viability under serum-free culture condition (Figure 10A and 10B) [5]. However, whatever in physiological or pathological conditions *in vivo*, it is impossible for vascular endothelial cells to be alive without serum; even in aging state *in vivo*, endothelial cells are still in the condition containing serum. Therefore, we hold the opinion that it was not convincing that cells were cultured in serum-free medium to observe the effects of GDF11. Serum-free culture condition is usually used to eliminate interference from the cytokines in serum. For example, Katsimpardi L *et al*. studied the function of GDF11 on brain capillary endothelial cells in serum-free basal medium for 6 days [5]. However, we found that the morphology of endothelial cell significantly altered under serum-free medium after 24h culturing, and the cell viability decreased when the cells were cultured in serum-free medium. Many works have confirmed that serum deprivation induced apoptosis in a variety of cells including endothelial cells [24-27]. Therefore, the medium containing serum was used to culture cells in our study. Under our experimental conditions, we found that GDF11 significantly activated smad2/3 and smad1/5/8 signals, indicating that the effects of GDF11 could be detected in the presence of serum.

In the present study, although both smad2/3 and smad1/5/8 signals were strongly activated by GDF11 in HUVECs, the cell proliferation and migration were not significantly increased or decreased. It was possibly due to the mutual effect of smad2/3 and smad1/5/8 signals. Most studies about GDF11 focused on the smad2/3 signals related to diverse physical and pathological processes [4, 28, 29]. As a member of BMP/TGF-β superfamily, we hypothesized that GDF11 should have the action with properties of both BMP and TGF-β family, and, indeed, we found that GDF11 activated not only smad2/3, but also smad1/5/8 in HUVECs. Both smad1/5/8 and smad2/3 signals are essential for endothelial function. Some reports suggested that smad2/3 signal was a positive role in endothelial cell function. For example, TGF-β promoted cell proliferation, migration and angiogenesis through smad3/NOX4/H_2_O_2_ signal [15]. However, it was also reported that the activation of smad2/3 signal could inhibit endothelial cell proliferation and cell migration [30]. Similarly, smad1/5/8 showed a controversial effect in endothelial cells. BMP9-induced the activation of ALK1 inhibited endothelial proliferation, migration and angiogenesis [31] and TGF-β-induced activation of ALK1 promoted the endothelial cell proliferation and migration [32]. Meanwhile, the smad2/3 and smad1/5/8 signals interacted on each other. TGF-β-induced ALK1 signal inhibited TGF-β-induced ALK-5 signal in the regulation of endothelial cell behavior [30]. In the course of differentiation of endothelial cells from bone marrow mesenchymal stem cells, smad3 promoted differentiation at an early phase, suppressed differentiation later and the activation of smad1/5/8 at a low level can promote differentiation [33]. Collectively, there existed a balance between smad2/3 and smad1/5/8 signals. Besides the canonical smad signal, GDF11 also activated JNK and AMPK signals. MitoTEMPO, which is a mitochondrial-targeted ROS inhibitor, restored GDF11-induced JNK and AMPK activation, indicating that GDF11-induced JNK and AMPK activation was secondary to ROS.

GDF11 induced an increase of NOX4 expression in HUVECs. NOX4 was reported to generate O^2−^ and H_2_O_2_, which could increase cell viability and migration of endothelial cell at a lower dose and induce endothelial apoptosis at a higher dose [20]. Thus, with the increase of NOX4 expression in HUVECs, ROS generation would gradually increase in the cells, which was similar to the treatment of tert-Butyl hydroperoxide with different concentrations (Figure 6A). However, it was notable that, although GDF11 slightly decreased cell viability, it did not induce cell death as demonstrated by live and dead cell staining assay (Figure 6C). Besides, whatever the increase of cell viability for short time treatment or decrease of cell viability for long time treatment, the difference was meaningless from the professional point of view.

In conclusion, we found GDF11 activated both smad1/5/8 and smad2/3 signals in HUVECs but showed no significant effect on cell migration and proliferation. Comparing with TGF-β1, the effect of GDF11 was also not unique. The present work suggests that the notion of GDF11 as a rejuvenation-related factor for endothelial cells needs to be cautious.

## MATERIALS AND METHODS

### Agents

Human umbilical vein endothelial cell (HUVEC) was purchased from Bioleaf (Shanghai, China). Dulbecco's modified Eagle's medium (DMEM) was purchased from Hyclone (Logan, Utah, USA). Fetal bovine serum (FBS) was purchased from Biological Industries (Beit Haemek, Israel). Recombinant human GDF-11 and recombinant human/mouse/rat GDF-11 were obtained from PeproTech and R&D Systems, respectively. Anti-NOX4, anti-smad1/5/8 and smad3 inhibitor SIS3 were purchased from Santa Cruz (Eugene, OR, USA). Anti-p-eNOS (Ser1177), anti-eNOS, anti-p-smad1(Ser463/465)/smad5(Ser463/465)/smad8(Ser426/428), anti-p-smad3(Ser423/425), anti-smad3, anti-p-smad2(Ser465/467), anti-smad2, anti-smad2/3, anti-p-Akt(Ser473), anti-p-Akt(Thr308), anti-Akt, anti-p-p38(Thr180/Tyr182), anti-p38, anti-p-JNK(Thr183/Tyr185), anti-JNK, anti-p-Erk1/2(Thr202/Tyr204), anti-Erk1/2, anti-p-AMPKα(Thr172), anti-AMPKα were obtained from Cell signal technology(Beverly, MA, USA). Anti-actin was obtained from ZSGB-BIO(Beijing, China). IRDye 800CW goat anti-mouse and IRDye 800CW goat anti-rabbit were purchased from Licor (Lincoln, Nebraska, USA). MitoTEMPO, Type IA collagenase and tert-Butyl hydroperoxide solution were purchased from Sigma-Aldrich (St. Louis, MO, USA). CFSE cell proliferation kit was obtained from Beyotime (Shanghai, China). LIVE/DEAD○RRViability/Cytotoxicity Assay Kit was obtained from Invitrogen (Eugene, OR, USA). MTT and dimethyl sulfoxide were obtained from Amresco (Solon, OH, USA). DMSO (dimethyl sulfoxide) was as solvent for stocking solution and its final concentration was limited within 4%_°_, which showed no effect on cell viability. Unless otherwise stated, GDF11 purchased from PeproTech was used.

### HUVEC cell culture

HUVEC cells were cultured in Dulbecco's modified Eagle's medium supplemented with 10% fetal bovine serum, 100 units/ml penicillin and 100 μg/ml streptomycin at 37°C, 5%CO_2_. The time of treatment and concentration of agents were shown in Figures and/or corresponding Figure legends.

### Primary rat aortic endothelial cell

Adult Sprague Dawley rats were anesthetized with pentobarbital sodium by intraperitoneal injection. Heparin (100units/mL) in phosphate buffer saline (PBS) was infused into heart from the view of the left ventricle and the abdominal aorta was cut off in order to remove the blood of vessel. Then the aorta was dissected in PBS containing 1% penicillin-streptomycin and aorta was incubated in collagenase type I solution for 10min at 37°C. Endothelial cells were collected by centrifugation, resuspended in 20% FBS-DMEM, and then endothelial cells were cultured. The detailed procedure was described [34]. All procedures involving animals and their care were approved by the Institutional Animals Care and Use Committee of Harbin Medical University, PR China.

### Western blot analysis

Western blot analysis was performed as described in our previous work [35, 36]. Cells were lysed with RIPA buffer containing 1% protease inhibitor, 10% phosphatase inhibitor and centrifuged at 13500r for 15min at 4°C. Then the supernatants were collected and the protein concentrations were determined by BCA assay kit (Beyotime, China). The protein was applied to 8% to 12% SDS-PAGE gels, transferred to nitrocellulose membranes. After incubation with the proper primary and secondary antibodies, Western blot bands were quantified by using Odyssey infrared imaging system (LI-COR) and Odyssey v3.0 software.

### MTT assay

Cell viability was assessed by MTT assay (tetrazolium bromide reduction) to measure mitochondrial succinate dehydrogenase activity in living cells. The assay is dependent on the ability of viable cells to metabolize a water-soluble tetrazolium salt into a water-insoluble formazan product. Cells were trypsinized and seeded in the 96-well plate. After adherence, complete medium was replaced with basal medium for 12 hours. The time of treatment and concentration of agents were shown in Figures and/or corresponding Figure legends.

### Cell wound healing assay

The wound-healing assay was used to determine cell migration ability. HUVECs were collected and sub-cultured with DMEM containing 10% FBS in six-well plate. A scratch was made by using a pipette tip on a uniform layer of cells. The scratch was washed with PBS twice to remove detached cells. The images were monitored at 0h, 6h and 24h for HUVECs and 0h, 18h, 24h and 40h for RAECs after scratching at the same area by taking photos with microscope at 4×10 magnification to measure the width of wound. The wound widths of different areas at different time points were measured with Image J software.

### Cell proliferation assay

Cell proliferation was measured by carboxyfluorescein diacetate succinimidyl ester (CFSE) staining. Endothelial cells were collected and suspended with DMEM containing 10% FBS, and then immediately labeled with CFDA SE(5μM) in the same volume under the condition of 37°C, 5%CO_2_ for 10 min. The staining was terminated by 40% volume precooling FBS for 10 min. Suspension cells were centrifuged at 1000 rpm for 5 min. The supernatant was discarded and washed by PBS buffer twice. Re-suspended endothelial cells were cultured in DMEM with 10% FBS under 37°C, 5%CO_2_ for 24h. The cells were collected by centrifuging at 1000rpm for 5 min, washed by PBS buffer and re-suspended by PBS buffer. The samples were filtered by 200 mesh strainer and analyzed by flow cytometry (excitation: 492 nm; emission: 517 nm). The cell proliferation is negatively correlated to the intensity of fluorescence.

### Live and dead cell staining

The cells were seeded in the six-well plate at a density of 3.75×10^4^/mL. After adherence, complete medium was replaced with basal medium for 12 hours. The time of treatment and concentration of agents were shown in Figures and/or corresponding Figure legends. Then the cells were washed with PBS for three times and stained according to the manufacturer's instructions. The labeled cells were photographed under a fluorescence microscope. The live cells were stained with calcein AM in green, and the dead cells were stained with ethidium homodimer-1 in red.

### Data analysis

Data are presented as mean±SEM. Significance was determined by using Student t test or one-way ANOVA, followed by Holm-Sidak post test. *P* < 0.05 was considered significant.
