# Correction: GDF11/BMP11 activates both smad1/5/8 and smad2/3 signals but shows no significant effect on proliferation and migration of human umbilical vein endothelial cells

**DOI:** 10.18632/oncotarget.10664

**Published:** 2016-07-18

**Authors:** Yong-Hui Zhang, Feng Cheng, Xue-Ting Du, Jin-Lai Gao, Xiao-Lin Xiao, Na Li, Shan-Liang Li, De-Li Dong

Present: Due to an error made by the authors while summarizing relevant publications, reference [5] was cited with a notice that serum-free cell culture condition was used.

Corrected: after discussing the matter with the authors of reference [5], authors of this study wish to correct the citation by stating that the culture medium with 5% serum and additional VEGF and EGF was used for endothelial cell culture in reference [5]. Authors sincerely apologize for this mistake.

Original article: Oncotarget. 2016; 7(11): 12063-74. doi: 10.18632/oncotarget.7642.

